# Frequent Use of Paracetamol and Risk of Allergic Disease Among Women in an Ethiopian Population

**DOI:** 10.1371/journal.pone.0022551

**Published:** 2011-07-21

**Authors:** Alemayehu Amberbir, Girmay Medhin, Charlotte Hanlon, John Britton, Andrea Venn, Gail Davey

**Affiliations:** 1 School of Public Health, Addis Ababa University, Addis Ababa, Ethiopia; 2 Division of Epidemiology and Public Health, University of Nottingham, Nottingham, United Kingdom; 3 Aklilu Lemma Institute of Pathobiology, Addis Ababa University, Addis Ababa, Ethiopia; 4 Department of Psychiatry, Addis Ababa University, Addis Ababa, Ethiopia; Ulm University, Germany

## Abstract

**Introduction:**

The hypothesis that paracetamol might increase the risk of asthma and other allergic diseases have gained support from a range of independent studies. However, in studies based in developed countries, the possibility that paracetamol and asthma are associated through aspirin avoidance is difficult to exclude.

**Objectives:**

To explore this hypothesis among women in a developing country, where we have previously reported aspirin avoidance to be rare.

**Methods:**

In 2005/6 a population based cohort of 1065 pregnant women was established in Butajira, Ethiopia and baseline demographic data collected. At 3 years post birth, an interview-based questionnaire administered to 945 (94%) of these women collected data on asthma, eczema, and hay fever in the past 12 month, frequency of paracetamol use and potential confounders. Allergen skin tests to *Dermatophagoides pteronyssinus* and cockroach were also performed. The independent effects of paracetamol use on allergic outcomes were determined using multiple logistic regression analysis.

**Findings:**

The prevalence of asthma, eczema and hay fever was 1.7%, 0.9% and 3.8% respectively; of any one of these conditions 5.5%, and of allergen sensitization 7.8%. Paracetamol use in the past month was reported by 29%, and associations of borderline significance were seen for eczema (adjusted OR (95% CI) = 8.51 (1.68 to 43.19) for 1–3 tablets and 2.19 (0.36 to 13.38) for ≥4 tablets, compared to no tablets in the past month; overall p = 0.055) and for ‘any allergic condition’ (adjusted OR (95% CI) = 2.73 (1.22 to 6.11) for 1–3 tablets and 1.35 (0.67 to 2.70) for ≥4 tablets compared to 0 in the past month; overall p = 0.071).

**Conclusions:**

This study provides further cross-sectional evidence that paracetamol use increases the risk of allergic disease.

## Introduction

There is now increasing and convincing epidemiological evidence from a range of independent studies implicating paracetamol use in the etiology of asthma and other allergic disease[1]–[3]. Previously our group has reported a dose-response relationship between paracetamol intake and self-reported wheeze, rhinitis and eczema in an Ethiopian population in which confounding by indication (implying that the drug might be given for symptoms of the disease or other conditions) was unlikely to play a role [4]; [5]. The multicountry ISAAC phase three study reported an association of paracetamol use in the first year of life with current wheeze, symptoms of rhinoconjunctivitis and eczema in a very large sample of children from 31 countries[6]. Paracetamol use was similarly associated with the risk of severe asthma symptoms with population-attributable risk estimates ranging from 22% to 38% [6]. The adverse effect of paracetamol on asthma has also been reported with exposure in *utero* [7] and during infancy,[5]; [8] in childhood[6]; [9] and adults [1]; [3]; [4]; [10]–[14].

The observation that higher paracetamol use may explain the increasing prevalence of asthma in English-speaking countries[13]–[15] and other regions of the world[6]; [9] is intriguing and suggests paracetamol as a putative risk factor for the development of asthma[2]. Whilst evidence is lacking on effects of therapeutic doses of paracetamol, it is biologically plausible that paracetamol may be involved in the etiology of asthma through glutathione depletion in the lung and reduced antioxidant capacity[16]; [17]. This depletion may also cause a shift from Th_1_ to Th_2_ cytokine production favoring allergic disorders[17]. Alternatively, paracetamol might influence COX-2 activity and production of prostaglandins E_2_ [15], which in turn favours a T-helper type-2 (Th2) immune dominance. Limited evidence also exists for pro-inflammatory effects of therapeutic doses of paracetamol through stimulation of the transient receptor potential ankyrin-1 channel [18].

In studies based in developed countries, the possibility that paracetamol and asthma are associated through reverse causation and avoidance of aspirin in people with asthma is difficult to exclude. We have therefore explored this hypothesis in a population of Ethiopian women among whom we have previously established that awareness of the potential dangers of aspirin use in asthma is very low and systematic avoidance of aspirin by individuals with allergic symptoms is remarkably rare[4].

## Methods

### Study area

The Butajira Rural Health Program (BRHP), cantered on the Butajira Demographic Surveillance Site (DSS), is located in southern Ethiopia, 130 km away from the capital Addis Ababa. It contains a dynamic cohort with an established data collection system covering more than 40,000 people living in 10 administrative areas (one urban and nine rural), details of which have been described elsewhere.[19] The DSS population contains 13,268 women of reproductive age[19]. A previous study in the source population reported the prevalence of paracetamol use to be 42% and showed no difference in use between urban and rural residents, between males and females or across educational levels[4]. More than three-quarters of the population knew paracetamol was different from aspirin[20].

### Study design and the birth cohort women

**Figure 1:**
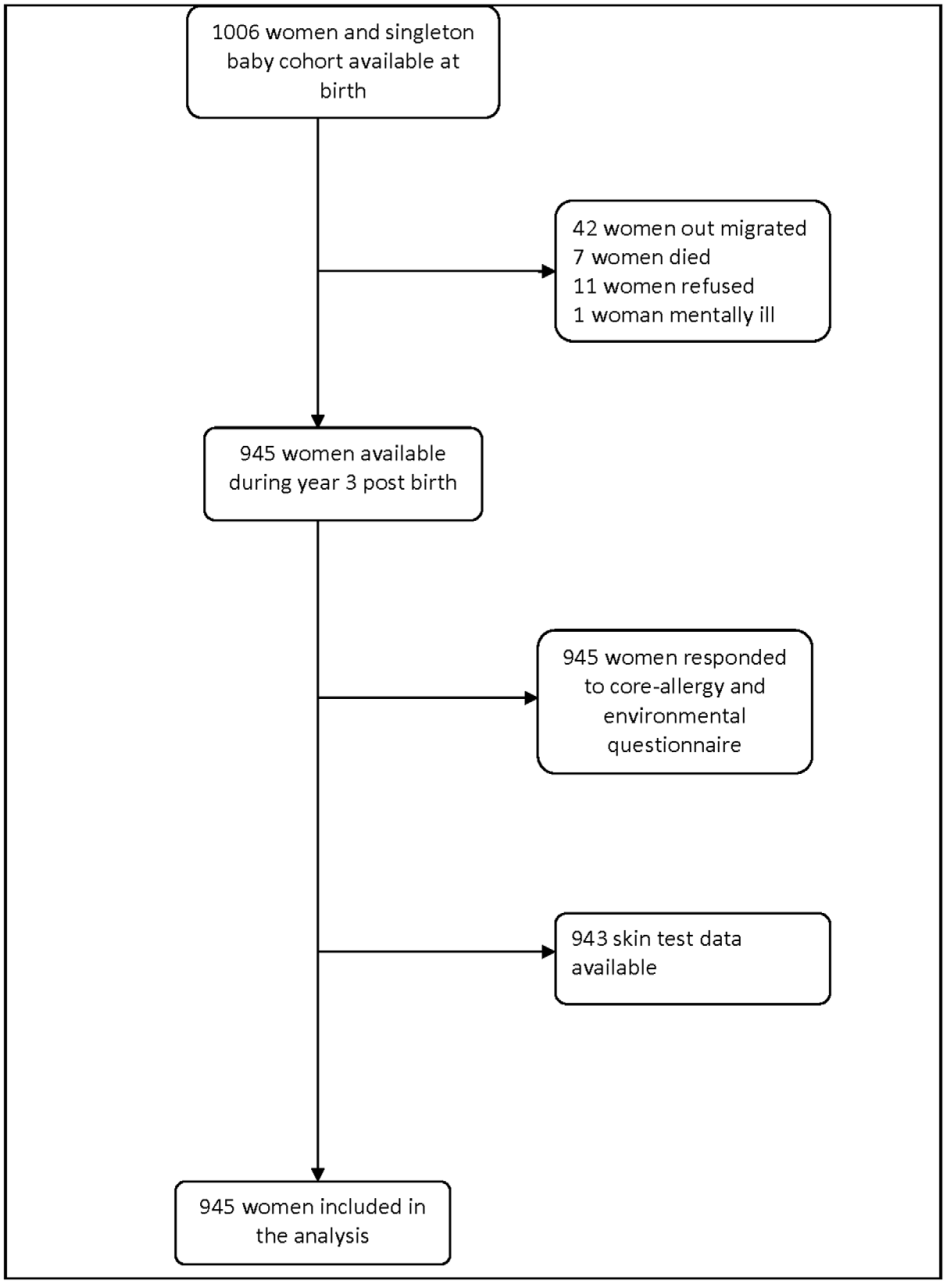
The birth cohort of women.

The birth cohort we report on is nested in the BRHP, and was established between July 2005 and February 2006. Full details have been described elsewhere[5]; [21]; [22]. In brief, pregnant women aged 15 to 49 years and in their third trimester were identified by the BRHP enumerators in the course of quarterly surveillance interviews and 1065 (86% of eligible) women were recruited in the birth cohort. Non-participating women did not differ from participating women in respect to socio-demographic characteristics, details of which were reported elsewhere[22]. Participating women gave birth to 1006 singleton live babies and have been followed in the cohort. The study reported here is a population based study of these birth cohort women three years post birth to investigate the association between paracetamol use and allergic diseases and sensitization (Fig 1).

### Data collection and measures

The Amharic translation of the International Union Against Tuberculosis and Lung Disease (IUALTD) bronchial symptoms questionnaire was administered to each woman. These measurements have been used previously in the same setting[4] and validated against free-running exercise test or bronchodilator response to inhaled salbutamol[23]; and were administered by data collectors known to the women since the establishment of the birth cohort. Women's history allergic was measured by using three questions: asthma *(‘In the last 12 months have you had asthma?’),* hay fever *(‘In the last 12 months have you had problems with sneezing or running nose when not have cold or flu, or problems with itchy watery eyes?’)* and eczema *(‘In the last 12 months have you had an itchy skin condition affecting the skin creases (front of the elbow, behind the knees, the front of the ankles, around the neck, or around the eyes?’).* We measured allergen skin sensitization to *Dermatophagoides pteronyssinus* and cockroach allergen (*Blattella germanica*) (Biodiagnostics, Upton-upon-Severn, UK) using skin-prick lancets on the palmar surface of the forearm of each woman. Glycerol saline and histamine dihydrochloride were used as negative and positive controls, respectively. To determine frequency of paracetamol use, women were asked "Have you taken any paracetamol in the last year?" and, if they responded yes, further asked "How many tablets of paracetamol have you taken in the last month?"

Questions on potential confounders including area of residence (urban/rural), age of the women, education and occupation of the women, type of roof and wall, cooking site, domestic fuel use, presence of animals in the compound, insecticide use, second hand smoke (the prevalence of women's smoking is very rare in this population), and sanitation were also included.

### Data analysis

Questionnaire and skin test data were double-entered into EpiData version 3.1 (EpiData Denmark). The datasets were cleaned, coded, and merged ready for analysis using Stata 11 (Statacorp, College Station Texas, USA). The primary outcome variables were asthma, hay fever, eczema and allergic sensitization to *D. pteronyssinus* and/or cockroach allergen. A positive test was defined as an average of two perpendicular wheal diameters, one of which was the maximum measurable diameter, of at least 3 mm greater than the saline control response. Sensitization to either *D. pteronyssinus* or cockroach allergen was defined as ‘any sensitization’ and a positive response to one or more of asthma, hay fever and eczema was defined as ‘any allergic condition.’ Paracetamol use in the past month was categorized as ‘none’, ‘1–3 tablets’ and ‘≥4 tablets’ similar to our previous paracetamol dose categories reported elsewhere[4]. The univariate association between paracetamol use and each outcome variable was assessed by computing odds ratios (ORs) and associated 95% confidence intervals (CIs) using binary logistic regression analysis. The independent effects of paracetamol use on the allergic outcomes were determined using multiple logistic regression analysis controlling for age of the women, urban and rural area of residence and women education. We have further explored the impact of controlling for other potential confounders listed in Table 1.

**Table 1:** Use of paracetamol in the past month by demographic and life style factors (N = 945).

Variables	N (%)	Paracetamol use in the past monthn (%)	P-value
Area of residence			
Urban	117 (12.4)	41 (35.0)	0.110
Rural	828 (87.6)	231 (27.9)	
Age of the women			
15–24	357 (37.8)	98 (27.5)	0.476
25–34	445 (47.1)	127 (28.5)	
35–44	143 (15.1)	47 (32.9)	
Education of the women			0.451
No formal education	758 (80.2)	214 (28.2)	
Formal education	187 (19.8)	58 (31.0)	
Occupation of the women			0.363
Housewife	791 (83.7)	223 (28.2)	
Any other job*	154 (16.3)	49 (31.8)	
Type of roof			0.246
Thatched	678 (77.5)	188 (27.7)	
Corrugated sheet	197 (22.5)	63 (32.0)	
Cooking site			0.092
Inside	706 (74.7)	193 (27.3)	
Outside	239 (25.3)	79 (33.1)	
Indoor kerosene use			0.574
Yes	55 (5.8)	14 (25.5)	
No	890 (94.2)	258 (29.0)	
Presence of animal			0.818
Yes	602 (63.8)	175 (29.1)	
No	342 (36.2)	97 (28.4)	

*Farm, trade and profession related and daily labourer.

### Study power

For an outcome with approximately 6% prevalence, our sample of 945 women provided 80% power at 95% significance level to detect an odds ratio of 2.2, for use of paracetamol ≥1 tablets/month compared with none.

### Ethics statement

The study was approved by the ethics committee of Nottingham University, United Kingdom and the ethics committee of the Ethiopian Science and Technology Ministry. Written, informed consent was obtained from all participants in keeping with requirements of the Ethiopia ethics committee.

## Results

### Demographics of the cohort women

Nine hundred forty five (94%) women in the cohort were followed up and responded to the core allergy and environmental questionnaire, and 943 (99.8%) provided skin test data. The majority of them were rural dwellers (88%) and nearly half aged between 25–34 years (Table 1). Only 20% of the women had any formal education and over 80% were housewives (Table 1). Most lived in houses with thatched roof (78%), and kept animals inside overnight (64%; Table 1).

### Prevalence of paracetamol use in women

Paracetamol had been used in the last year by 470 (49.7%) women and in the last month by 272 (28.8%) women. In the last month, 8.7% of women reported taking 1–3 tablets and 20.1% of women 4 or more tablets. Use in the past month was unrelated to age and education of the women, area of residence, roof type, site of cooking, domestic fuel use or presence of animals inside the house (Table 1).

### Prevalence of allergic diseases in women

Asthma was reported in 1.7% (16/945) of women, hay fever in 3.8% (36/945), and eczema in 0.9% (8/945), with a combined prevalence of ‘any allergic condition’ of 5.5% (52/945) (Table 2). The prevalence of *D. pteronyssinus* and cockroach sensitization was 5.6% (53/943) and 2.8% (26/943) respectively, and sensitization to either allergen was found in 7.8% (74/943) of the women (Table 2). Self-reported asthma, hay fever and eczema were all more prevalent in urban than rural women, significantly so for asthma and hay fever (Table 2). There was no significant difference in allergic sensitization by area of residence (Table 2).

**Table 2:** Prevalence of allergic conditions and sensitization among women by area of residence.

Outcome	Overalln (%)	Urbann (%)	Ruraln (%)	Crude OR(95% CI)	p-value
Asthma (N = 945)	16 (1.7)	9 (7.7)	7 (0.9)	9.77 (3.57,26.78)	<0.001
Hay fever (N = 945)	36 (3.8)	13 (11.1)	23 (2.8)	4.38 (2.15,8.90)	<0.001
Eczema (N = 945)	8 (0.9)	2 (1.7)	6 (0.7)	2.38 (0.48,11.95)	0.291
Any allergic conditions*(N = 945)	52 (5.5)	19 (16.2)	33 (4.0)	4.67 (2.56,8.53)	<0.001
*D. pteronyssinus* (N = 943)	53 (5.6)	8 (6.8)	45 (5.5)	1.27 (0.58,2.77)	0.542
Cockroach (N = 943)	26 (2.8)	2 (1.7)	24 (2.9)	0.58 (0.14,2.49)	0.465
Any sensitization^†^ (N = 943)	74 (7.8)	10 (8.6)	64 (7.7)	1.12 (0.56,2.24)	0.758

*one or more of asthma, eczema and hay fever.

¥Sensitization to either *D*. *pteronyssinus* or cockroach allergen.

### Effects of paracetamol on allergic diseases in women

Table 3 shows that both before and after control for confounders, paracetamol use was associated with a non-significant increased risk of asthma, and hay fever, and a borderline statistically significance increased risk of eczema (ORs [95% CIs] adjusted for age, area of residence and education level = 8.51 [1.68 to 43.19] for 1–3 tablets and 2.19 [0.36, 13.38] for ≥4 tablets compared with non-use; overall p = 0.055). The association between paracetamol and ‘any allergic condition’ was also of borderline statistical significance (p = 0.07) with the greatest risk in the women taking 1–3 tablets in the past month (adjusted OR [95% CIs] = 2.73 [1.22 to 6.11] compared to none; Table 3). No significant association between paracetamol use and allergic sensitization was seen (Table 3). Further control for other potential confounders did not materially alter the magnitude of the odds ratios presented.

**Table 3:** OR for asthma, eczema, hay fever and sensitization in relation paracetamol use in the past month in women.

Outcome	Paracetamol use in past month	Yesn (%)	CrudeOR (95%CI)	AdjustedOR† (95% CI)	P-value
Asthma(N = 945)	None	9 (1.3)	1	1	0.625‡
	1–3 tablets	2 (2.4)	1.84 (0.39,8.69)	1.76 (0.36,8.62)	0.364¶
	≥4 tablets	5(2.6)	1.99 (0.66,6.02)	1.64 (0.52,5.14)	
					
Eczema(N = 945)	None	3 (0.5)	1	1	0.055‡
	1–3 tablets	3 (3.7)	8.48 (1.68,42.74)	8.51 (1.68,43.19)	0.225¶
	≥4 tablets	2 (1.1)	2.38 (0.39,14.32)	2.19 (0.36,13.38)	
					
Hay fever(N = 945)	None	23 (3.4)	1	1	0.526‡
	1–3 tablets	5 (6.1)	1.84 (0.68,4.97)	1.86 (0.67,5.12)	0.775¶
	≥4 tablets	8 (4.2)	1.24 (0.55,2.82)	1.03 (0.44,2.41)	
					
Any allergic conditions*(N = 945)	None	30 (4.5)	1	1	0.071‡
	1–3 tablets	9 (11.0)	2.64 (1.21,5.78)	2.73 (1.22,6.11)	0.225¶
	≥4 tablets	13 (6.8)	1.57 (0.80,3.08)	1.35 (0.67,2.70)	
					
*D. pteronyssinus* sensitization(N = 943)	None	37 (5.5)	1	1	0.481‡
	1–3 tablets	7 (8.5)	1.60 (0.69,3.72)	1.60 (0.69,3.72)	0.856¶
	≥4 tablets	9 (4.8)	0.86 (0.41,1.81)	0.85 (0.40,1.79)	
					
Cockroach sensitization(N = 943)	None	18 (2.7)	1	1	0.903‡
	1–3 tablets	2 (2.4)	0.91 (0.21,3.99)	0.91 (0.21,4.02)	0.708¶
	≥4 tablets	6 (3.2)	1.19 (0.47,3.04)	1.22 (0.48,3.13)	
					
Any sensitization¥ (N = 943)	None	50 (7.4)	1	1	0.564‡
	1–3 tablets	9 (11.0)	1.54 (0.73,3.25)	1.53 (0.72,3.24)	0.668¶
	≥4 tablets	15 (7.9)	1.07 (0.59,1.95)	1.07 (0.59,1.96)	

† OR adjusted for age of the women, area of residence and women education.

*include asthma, eczema and hay fever.

¥ Sensitization to either *D*. *pteronyssinus* or cockroach allergen.

‡ Likelihood ratio test.

¶ P value for trend.

## Discussion

In this cohort of developing country women, we have demonstrated borderline significant positive associations between paracetamol use and reported eczema and ‘any allergic condition’, independent of age, social class and numerous other potential confounders. Whilst we found no significant associations between women's use of paracetamol and sensitization, the observed effects were in the expected direction.

We have made use of a cohort of women in a resource-poor setting to measure allergic outcomes and paracetamol exposure in a population which can distinguish paracetamol from other analgesics. Although we have not collected information on aspirin use, our previous nested study showed that aspirin avoidance amongst those with allergic diseases is rare[4]. Furthermore, we have previously shown that use of paracetamol for treating the symptoms of asthma and other allergic conditions is also rare [4]; [20] making reverse causation an unlikely alternative explanation for our current findings. Confounding by use of non-steroidal anti-inflammatory drugs (NSAIDs) such as ibuprofen is also a potential issue [24], and previous studies have accounted for these confounders, as well as aspirin use, in their analyses[7]; [10]; [12]; [14]; [24]. However, this is an unlikely source of bias in our study since NSAIDs were not readily available or affordable in this rural community. We acknowledge that residual confounding by a factor not measured is difficult to exclude. A further strength of this study is the inclusion of a large number of potential confounders like social advantage that might have been linked with prescription or over-the-counter use of paracetamol. However, we found that none of our markers of socioeconomic status were associated with women's use of paracetamol, which make this unlikely to explain our findings.

Whilst our sensitization outcomes were measured objectively and based on domestic allergens previously found to be common in Ethiopia,[25] our measures of asthma, hay fever and eczema were based on self report and hence susceptible to reporting or information bias. Of particular concern was misclassification of scabies or other skin condition as eczema. However, we have previously shown that the short-term repeatability of wheeze and asthma questionnaire in a similar setting is generally good, even though validity of these symptoms in relation to exercise test or bronchodilator challenge is limited[23]. The outcome measures used focused on asthma rather than wheeze, primarily because our previous validation work in the same setting and in a similar age group showed short-term Kappa was higher for asthma than wheeze responses[23]. The low prevalence of our allergic symptoms and sensitization outcomes, however, has limited the power of the study which was partly reflected by the wide confidence interval around the risk estimates.

Paracetamol use was also ascertained by self report, but recall error was minimized by the fact we based our exposure variable on dose of paracetamol in the last month, and also medication strips or other containers were cross-checked where possible. Furthermore, we have previously established that adults in Butajira are able to differentiate paracetamol from other analgesics[20]. Since there is no reason that allergic subjects would be more or less likely to report paracetamol use, any reporting error is likely to be non-differential and would therefore result in our odds ratios being biased towards the null value.

Eczema and ‘any allergic condition’ were associated with paracetamol use. The risk of asthma, although not significantly associated with paracetamol exposure, was also elevated among paracetamol users. Our finding is congruent with several earlier epidemiological studies indicating an association between paracetamol use and allergy in women[10] and adults[1]; [4]; [11]–[14]. In 2005, we reported similar significant and consistent dose-dependent associations between paracetamol use and eczema, rhinitis, shortness of breath and sensitization to cockroach allergens for which alternative explanations were unlikely[4]. However, this earlier study, like the current one, was unable to show a significant relation between paracetamol use and self-reported asthma which may reflect under-diagnosis of asthma in this rural setting[4]. The lack of dose-response relationship in this study may reflect the relatively small numbers of women in each subgroup or errors in reporting doses, leading to misclassification of dose category. The increased risk of eczema in this cohort of women suggests that risk is not restricted to the airways and is consistent with the findings of the ISAAC multicountry study[6]. This would fit with mechanisms that suggest that paracetamol predisposes to glutathione depletion which may influence the promotion of atopy through increased production of Th2 cytokine responses[17]. A non-significant positive association was also been seen between women's paracetamol use and any skin sensitization. This is consistent with the European Community Respiratory Health Survey which reported a non significant tendency to higher prevalence of atopy (defined as specific IgE titre of >0.35 KU. L^−1^ to four allergen tested) among adults in centres with higher paracetamol sales[13]. The authors suggested antioxidant depletion by paracetamol might influence allergic diseases at least in adults[13].

Among the observational studies in adults are two United States-based studies[10]; [11]. These studies showed an adverse role of paracetamol use on asthma, chronic obstructive lung disease (COPD), and decreased lung function[10]; [11]. The study by Barr *et al* [10] prospectively examined paracetamol use on new onset asthma in women and found an increased rate of new physician diagnosis of adult onset-asthma. The finding supports the hypothesis that population-level increases in asthma in the United States might be attributable partly to a shift from aspirin to paracetamol[15]. Further evidence comes from a United Kingdom-based case-control study[12] and the European Network case-control study,[14] both of which reported increased risk of asthma and other allergic disease morbidity with frequent use of paracetamol. Further support for this hypothesis came from a population-level ecological study in English-speaking countries[13]. Newson and colleagues in this study[13] took paracetamol sale as a proxy for frequent use, and demonstrated that sales at country level were associated with increased risk of asthma, eczema, rhinoconjunctivitis, and bronchial responsiveness.

In conclusion, this study shows a borderline significant association between paracetamol use and symptoms of eczema and ‘any allergic conditions’ in low-resource setting cohort of women in which we have previously reported that aspirin avoidance or bias in reporting exposure are unlikely to play a role. We have also reported similar adverse effects in an equivalent cohort of children in this population[5]. Together these findings add additional support to the paracetamol-allergy hypothesis and highlight the need for a randomized controlled trial.
